# Dynamic causal modelling of COVID-19 and its mitigations

**DOI:** 10.1038/s41598-022-16799-8

**Published:** 2022-07-20

**Authors:** Karl J. Friston, Guillaume Flandin, Adeel Razi

**Affiliations:** 1 The Wellcome Centre for Human Neuroimaging, University College London, London, UK; 2 Turner Institute for Brain and Mental Health and Monash Biomedical Imaging, Monash University, Clayton, Australia; 3 CIFAR Azrieli Global Scholars Program, CIFAR, Toronto, Canada

**Keywords:** Viral infection, Computational science

## Abstract

This technical report describes the dynamic causal modelling of mitigated epidemiological outcomes during the COVID-9 coronavirus outbreak in 2020. Dynamic causal modelling is a form of complex system modelling, which uses ‘real world’ timeseries to estimate the parameters of an underlying state space model using variational Bayesian procedures. Its key contribution—in an epidemiological setting—is to embed conventional models within a larger model of sociobehavioural responses—in a way that allows for (relatively assumption-free) forecasting. One advantage of using variational Bayes is that one can progressively optimise the model via Bayesian model selection: generally, the most likely models become more expressive as more data becomes available. This report summarises the model (on 6-Nov-20), eight months after the inception of dynamic causal modelling for COVID-19. This model—and its subsequent updates—is used to provide nowcasts and forecasts of latent behavioural and epidemiological variables as an open science resource. The current report describes the underlying model structure and the rationale for the variational procedures that underwrite Bayesian model selection.

## Introduction

Since the introduction of dynamic causal modelling for quantitative prediction of the COVID-19 coronavirus epidemic^1,2^, the structure of the model has been progressively optimised as new data become available. In this technical report, we describe the model’s structure and provide illustrative predictions of various outcomes at the time of submission (i.e., 6-Nov-2020) (Note: As noted in the Epilogue, this paper provides the motivation for—and account of—a particular instance of a DCM of COVID-19 at a specific point in time. Due to the nature of dynamic causal modelling, the optimal structure of the model necessarily changes as more data becomes available and model complexity increases. A weekly record of successive changes to the model can be found here^3^). These illustrative examples focus on identifying models with the greatest evidence and how this underwrites predictive validity.

Dynamic causal modelling (DCM) stands apart from most modelling in epidemiology by predicting *mitigated* outcomes—and quantifying the uncertainty associated with those outcomes. This stands in contrast to quantitative epidemiological forecasts that do not consider the effect of prevalence on sociobehavioural responses. Usually, these projections are over few weeks—and rest upon fitting curves to the recent trajectory of various data; e.g., Refs.^4,5^. In contrast, dynamic causal modelling considers what is most likely to happen, based upon a generative model that best explains all the data available. This mandates a model of sociobehavioural responses that mitigate viral transmission, such as social distancing, lockdown, testing and tracing, etc. In turn, this requires a detailed consideration of how various sorts of data are generated. For example, it has to model fluctuations in testing capacity and sampling bias due to people self-selecting when symptomatic. The advantage of this kind of modelling is that any data generated by the model can be used to inform the model parameters that underwrite fluctuations in latent states, such as the prevalence of infection. Latent states refer to those states of the population that cannot be estimated directly and have to be inferred from observable data.

Dynamic causal modelling focuses not on worst-case scenarios but on the most likely outcomes, given concurrent predictions of viral transmission, responses in terms of behavioural interventions and changes in the way that the epidemic is measured (e.g., confirmed cases, death rates, hospital admissions, testing capacity, etc.). Crucially, dynamic causal modelling brings two things to the table. The first is the use of variational procedures to assess the quality of—or evidence for—any given model. This means that the model adapts to the available data; in the sense that the best model is taken to be the model with the greatest evidence, given the current data. As time goes on, the complexity of the model increases, in a way that is necessary to explain the data accurately. Technically, log evidence (a.k.a., marginal likelihood) is accuracy minus complexity—and both are a function of the data^6^.

The second advantage of dynamic causal modelling is a proper incorporation of uncertainty in the estimation of conditional dependencies. In other words, it allows for the fact that uncertainty about one parameter affects uncertainty about another. This means dynamic causal models generally have a large number of parameters, such that the conditional uncertainty about all the parameters is handled together. This furnishes a model that is usually very expressive and may appear over-parameterised. However, by optimising the prior probability density over the model parameters, one can optimise the complexity (c.f., the effective number of parameters), using Bayesian model selection^7–9^. Note that the ability to pursue this form of structure learning rests on being able to estimate the model evidence or marginal likelihood, which is one of the primary *raisons d'être* for the variational procedures used in dynamic causal modelling^10–12^.

These potential advantages can be leveraged to model a large variety of data types, to fit an expressive model of epidemiological trajectories and, implicitly, produce posterior predictive densities over measurable outcomes. In other words, parameterising behavioural responses—such as social distancing—as a function of latent states, enables the model to guess how we will respond in the future, with an appropriate uncertainty. This is the basis of the predictions of mitigated responses above.

The remainder of this report provides a brief description of current predictions using 10 sorts of data. These posterior predictive densities are based upon the implementation of DCM for COVID-19 described in the Supplementary Information and detailed in the accompanying annotated MATLAB code (see software note).

## Dynamic causal modelling

The convolution, generative or forward model of epidemic data (here, from the United Kingdom) is based upon four factors, each of which corresponds to a distinct kind of latent state, each with a number of distinct levels. One of these factors (the *infection* factor) can be thought of as a conventional (SEIR) epidemiological model. The remaining factors are concerned with population fluxes and fluctuating contacts between people, who may or may not be affected, and the clinical progression of the infection that depends on—but is separate from—the infection factor. This allows for both symptomatic and asymptomatic clinical corollaries of infection. The final factor concerns testing. This is a key part of the generative model because it generates the data considered to be informative about the course of the epidemic.

**Figure 1:**
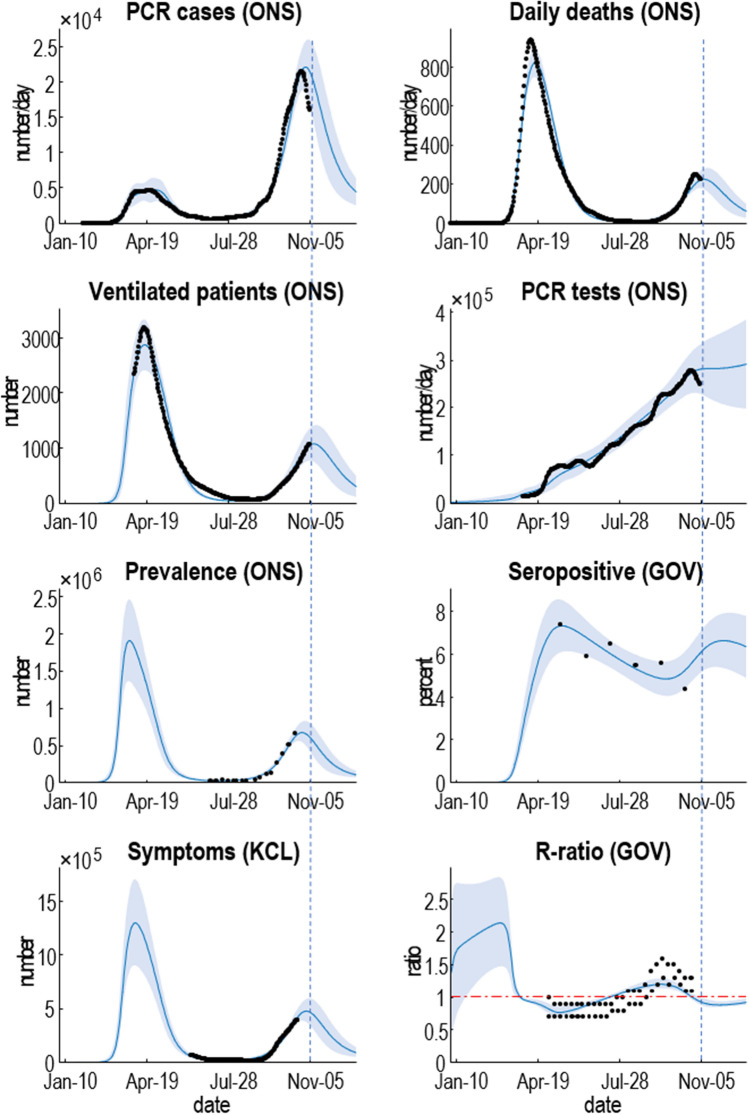
Posterior predictions of various outcome modalities, ranging from confirmed cases through to the reproduction ratio. The black dots correspond to smoothed empirical data from the publicly available sources listed in the main text. The blue lines and shaded areas correspond to the posterior expectation and 90% Bayesian credible intervals based upon the posterior predictive density. The vertical blue dashed line indicates the date at which the analysis was performed.

The particular DCM described in the Supplementary Information is based upon the original model of a single region^1^. It has subsequently been extended to deal with viral spread both within and between communities^2^. This was necessary to explain secondary waves^13^. A further difference between early applications of dynamic causal modelling, in this setting, and current applications is the use of multimodal data. The example in Fig. 1 uses 10 sources of data (see Supplementary Information for details):

- confirmed cases based upon PCR testing, as reported by specimen date
- daily deaths within 28 days of testing positive for COVID-19, reported by date of death
- critical care unit occupancy as measured by the number of patients requiring mechanical ventilation
- the number of PCR tests performed each day
- the number of people infected, based upon unbiased community surveys using PCR tests
- the percent of people who are seropositive, based upon unbiased community surveys using antibody tests
- the number of people reporting symptoms, as estimated by the COVID Symptom Study
- estimates of the reproduction ratio, issued by the government
- mobility, as measured by Department of Transport estimates of car use
- location, as estimated by Google's mobility data; i.e., workplace activity.

Note that some of these so-called data would be treated as estimates in conventional modelling, for example, the effective reproduction ratio. However, dynamic causal modelling treats these estimates as data features because they are based upon historical data. In other words, dynamic causal modelling generates the underlying reproduction ratio directly from latent states, such as the rate of change of prevalence of infection. This means it can then predict estimates based upon legacy data, under the assumption that there are random effects that accompany these conventional estimators.

Figure 1 shows the data (black dots) and predictions in terms of posterior expectations (blue lines) and associated 90% credible intervals (shaded areas). Here, we consider the first eight outcomes listed above. Note that the period over which data is available—and the time between observations—varies with different data types. However, because the generative model operates in continuous time, from the beginning of the outbreak into the future, all data points can be used. Here, we see that the first and secondary waves of confirmed cases show a marked asymmetry, with a much larger number of confirmed cases in the secondary wave. This is largely a reflection of the number of tests performed, as evidenced by the antisymmetric profile of daily deaths (averaged over seven days), which were predicted to peak at around 200 deaths per day (Note: Note that this prediction pertains to death by date, not by date reported, which has exceeded 400 on at least one day at the time of writing.) in early November 2020 (specifically, November 8). This pattern is reflected in the number of patients requiring mechanical ventilation, the estimated prevalence of infection from community surveys and the symptoms reported using the COVID Symptom Study^14^.

The model also shows a decline in seropositivity from about 7 to 5% at the time of writing, which is predicted to increase again over the next few weeks. The reproduction ratio started at over 2 and fell, during the first lockdown, to below one, dipping to a minimum of about 0.7 over the summer. After this, it rose to about 1.5 and fell below 1 in October. Note that the conventional estimates of the reproduction ratio—relative to the posterior expectation from DCM—are a slight overestimate. Crucially, the black dots in this figure (upper and lower confidence intervals, based upon consensus from the SPI-M: the UK Govt. Scientific Pandemic Influenza Subgroup on Modelling^15^) have been *shifted 2 weeks backwards* in time from their date of reporting. This is what is meant above by historical or retrospective estimates. In other words, the estimates of the reproduction ratio pertain to states of affairs a few weeks ago. At the time of writing, this was particularly relevant because a national lockdown had just been announced, with the aim of getting the reproduction ratio below one. According to this analysis, it was already below one at the time of the announcement^16^.

**Figure 2:**
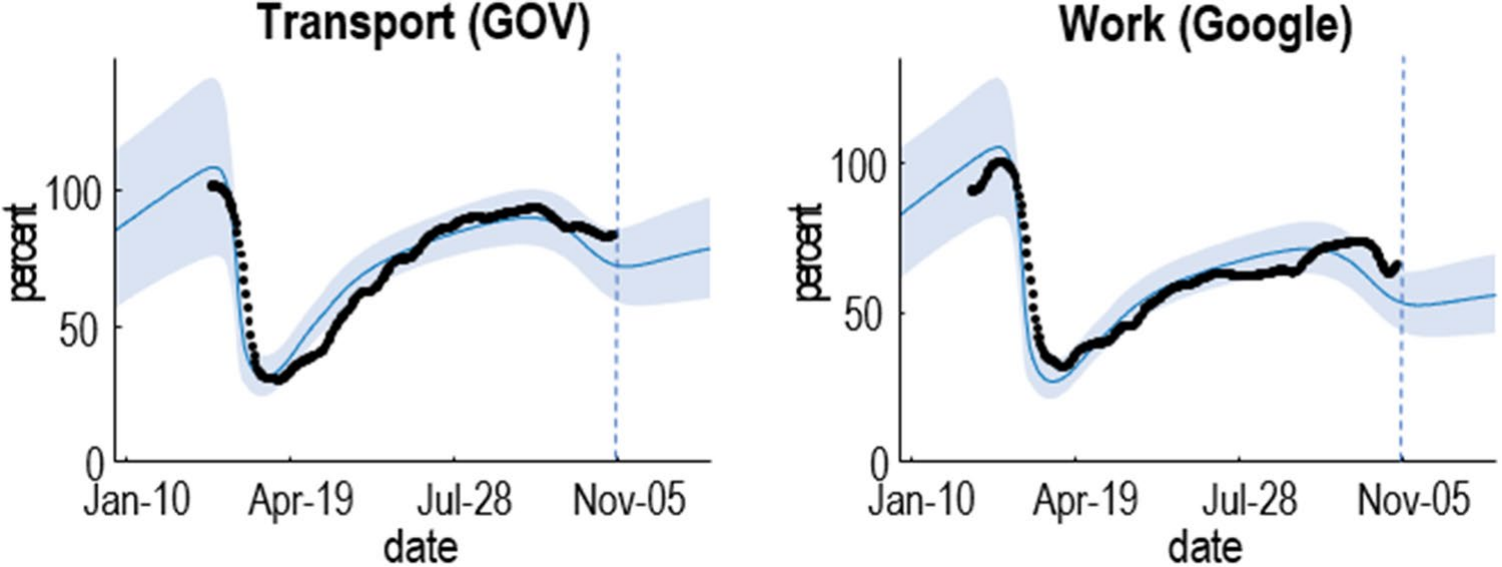
Posterior predictions for mobility. This figure uses the same format as Fig. 1. In this case, the data are expressed as a percentage of pre-pandemic values; namely, transport use and workplace activity, as estimated from Google mobility data. Please see main text for the data sources.

Figure 2 shows the equivalent results for mobility and location based upon Department of Transport and Google mobility data. It suggests that the national lockdown in spring reduced contact rates to about 25% of pre-COVID levels; after which they rose again slowly, until the resurgence of infections at the onset of the secondary wave. In Fig. 2, 100% refers to the pre-COVID mobility.

Uncertainty quantification is an inherent aspect of the data assimilation afforded by dynamic causal modelling. In other words, dynamic causal models are convolution models that use Bayesian or variational approaches to assimilate data—and the attending uncertainty. In this particular dynamic causal model, all the uncertainty resides in the model parameters, such as various rate constants and probabilities (see Table 1 and the Supplementary Information). This uncertainty is then propagated through to time-dependent latent states and, ultimately, the outcomes (denoted by the shaded confidence intervals in the figures above).

**Table 1:** Parameters of the dynamic causal model. Sources: Refs.^18,19^.

Number	Name	Description	Prior mean	Precision	Lower (95%)	Upper (95%)	Posterior mean	Lower (95%)	Upper (95%)
1	*N*	Population size (M)	66.65	Inf	66.65	66.65	66.65	66.65	66.65
2	*n*	Initial cases	1	0.25	0.037	26.8	1.04	0.41	2.6
3	*r*	Pre-existing immunity	0.1	16	0.066	0.15	0.047	0.032	0.069
4	*o*	Initially exposed	0.1	16	0.066	0.15	0.14	0.13	0.15
5	*out*	P (leaving home)	0.3	64	0.24	0.36	0.27	0.23	0.31
6	*sde*	Threshold: distancing	0.05	64	0.040	0.061	0.047	0.044	0.049
7	*qua*	Threshold: quarantine	0.005	64	0.0040	0.0061	0.0095	0.0083	0.010
8	*exp*	P (leaving area)	0.0005	16	0.0003	0.0007	0.0010	0.0009	0.0011
9	*cap*	CCU beds per person	0.00032	16	0.00021	0.00048	0.00057	0.00043	0.00076
10	*s*	Distancing sensitivity	2	64	1.6	2.4	2.4	2.2	2.7
11	*u*	Quarantine sensitivity	6	64	4.8	7.3	7.32	5.9	8.9
12	*c*	Mechanical sensitivity	1	64	0.81	1.2	0.79	0.65	0.96
13	*nin*	Contacts: home	2	64	1.6	2.4	1.0	0.88	1.3
14	*nou*	Contacts: work	64	64	52	78.6	81	70	94
15	*trm*	Transmission (early)	0.5	64	0.40	0.61	0.53	0.46	0.61
16	*trn*	Transmission (late)	0.5	64	0.40	0.61	0.27	0.23	0.31
17	*tin*	Infected period (days)	5	1024	4.7	5.2	4.9	4.7	5.1
18	*tcn*	Infectious period (days)	4	1024	3.7	4.2	3.4	3.3	3.6
19	*tim*	Loss of immunity (days)	256	64	208	314	222	208	237
20	*res*	Seronegative proportion	0.4	64	0.32	0.49	0.45	0.42	0.49
21	*tic*	Incubation period (days)	4	1024	3.7	4.2	3.0	2.8	3.1
22	*tsy*	Symptomatic period (days)	6	1024	5.6	6.3	6.7	6.5	7.0
23	*trd*	ARDS period (days)	11	1024	10.4	11.5	8.8	8.5	9.1
24	*sev*	P(ARDS|symptoms): early	0.005	1024	0.0047	0.0052	0.0047	0.0044	0.0049
25	*lat*	P(ARDS|symptoms): late	0.005	0.25	0.00018	0.13	0.0035	0.0030	0.0041
26	*fat*	P(fatality|ARDS): early	0.5	1024	0.47	0.52	0.44	0.42	0.46
27	*sur*	P(fatality|ARDS): late	0.5	0.25	0.018	13.4	0.31	0.25	0.38
28	*ttt*	FTTI efficacy	0.036	1024	0.034	0.037	0.037	0.035	0.039
29	*tes*	Testing: bias (early)	1	0.25	0.037	26.8	0.22	0.017	2.9
30	*tts*	Testing: bias (late)	4	0.25	0.14	107	5.2	4.8	5.7
31	*del*	Test delay (days)	4	1024	3.7	4.2	3.9	3.7	4.1
32	*ont*	Symptom demand	0.01	0.25	0.00037	0.26	0.0074	0.0045	0.012
33	*fnr*	False-negative rate	0.2	1024	0.18	0.21	0.20	0.19	0.21
34	*fpr*	False-positive rate	0.002	1024	0.0019	0.0021	0.0020	0.0019	0.0021
35	*lin*	Testing: capacity	0.01	0.25	0.00037	0.26	0.0065	0.0059	0.0071
36	*rat*	Testing: constant	48	0.25	1.7	1288	44	41	48
37	*ons*	Testing: onset	200	0.25	7.4	5367	203	194	213

Table 1 lists the parameters of this model, their priors and their posteriors based upon the data above. The free parameters are listed in the second column and their role in shaping the epidemiological dynamics is described in the Supplementary Information.

Although efficient, variational procedures are notoriously overconfident. They underestimate the uncertainty because of the way they handle conditional dependencies: see discussion and Ref.^17^. To compensate for this, the confidence intervals in the above figures have been inflated by multiplying the posterior standard deviation by a factor of eight. Crucially, these confidence intervals do not incorporate uncertainty about the structure or form of the model itself. In other words, although the model has been optimised to maximise model evidence, there is no guarantee that this is the best of all possible models, which it will almost certainly not be.

**Figure 3:**
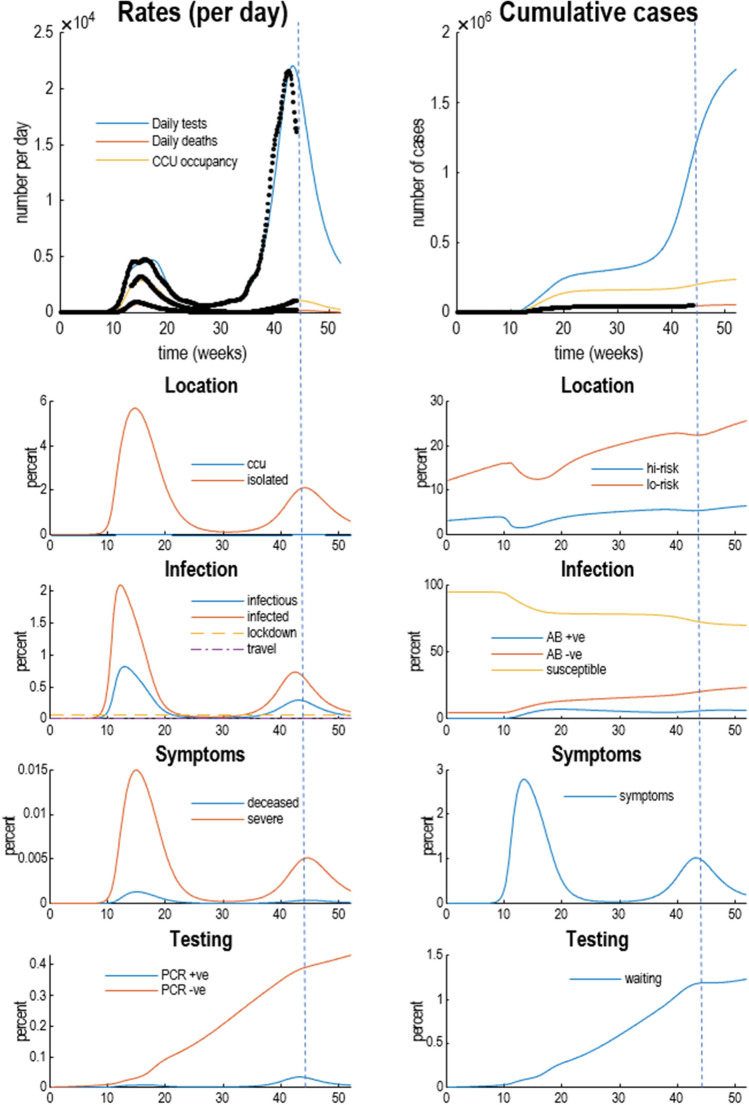
Posterior predictions and underlying latent states. The upper two panels show selected outcomes from the previous figures (black dots): here, daily confirmed cases using PCR testing, daily deaths, and critical care occupancy. The upper left panel shows the rates, while the upper right panel shows the cumulative totals. The remaining panels detail fluctuations in the latent states of the four factors. Each factor has two panels, showing each of the accompanying levels. For clarity, some levels have been omitted because the probabilities of being in any level—of any given factor—sum to one. For a more detailed explanation of what these latent states mean—and how they can be interpreted—please see the Supplementary Information.

Figure 3 shows the underlying latent states generating the predictions in Figs. 1 and 2. The upper two panels show three outcomes from the previous figures (black dots): daily confirmed cases using PCR testing, daily deaths, and critical care occupancy. The upper left panel shows the rates, while the upper right panel shows the cumulative totals. The remaining panels report the fluctuations in the latent states of the four factors. Each factor has two panels, showing each of the accompanying levels. For a more detailed explanation of what these latent states mean—and how they can be interpreted—please see the Supplementary Information.

The infection panel includes prior thresholds for restrictions on contacts within (lockdown) and between (travel) an *effective* or active population (broken lines). It is these thresholds that produce the periodic expression of secondary and subsequent waves following the initial outbreak. One key thing to note here is that, in this example, about 70% of the population remains susceptible to future infection (after January 2021). The remaining population are at low risk of coming into contact with the virus or have acquired an effective immunity; irrespective of whether they are seropositive or seronegative.

Table 1 provides a brief description of the parameters, their prior densities and the posterior density afforded by fitting the data in the figures above. The prior precision corresponds to the inverse variance of the log-transformed priors. Although the (nonnegative) scale parameters are implemented as probabilities or rates, they are estimated as log parameters. The prior means and ranges were based upon the above sources—and have been progressively optimised with successive versions of the model using Bayesian model reduction^8^. Note that some parameters have narrow (informative) priors, while others are relatively uninformed. The upper and lower ranges of the prior and posterior confidence intervals contain 90% of the probability mass. Note that these are probabilistic ranges, and the posterior estimates can exceed these bounds, if the data calls for it.

## Predictive validity

**Figure 4:**
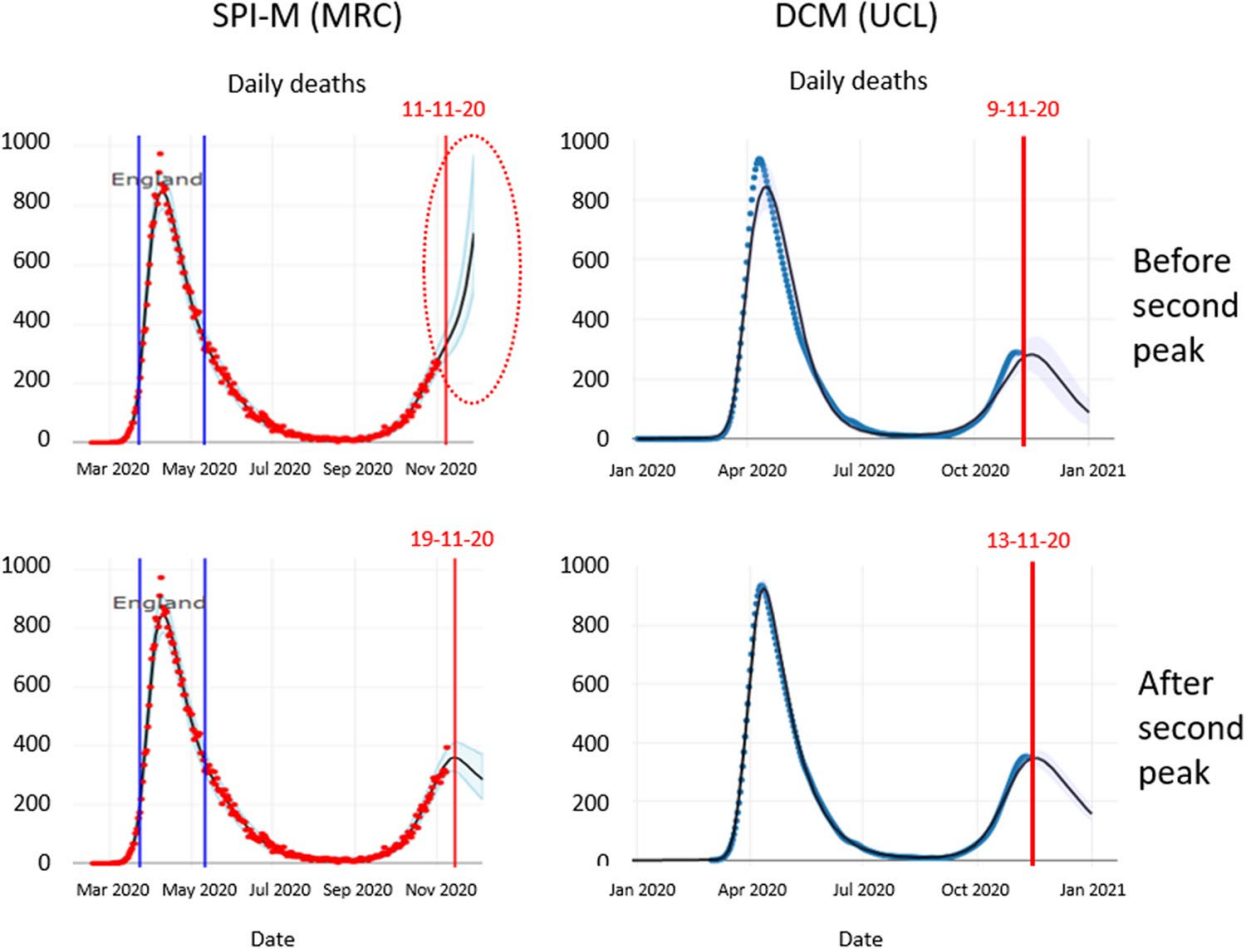
Predicting the second peak: The left panels show the forecasts from the MRC Biostatistics Unit at the University of Cambridge, UK, shortly before and after the peak of a second surge. These projections were taken as screen grabs from the dashboard on appropriate days. The right panels show the equivalent predictions using dynamic causal modelling. The top row shows the predictions before the second peak, while the bottom row shows the equivalent predictions a few days later. The dates are indicated by the vertical red lines. The red ellipse highlights an example of overfitting recent trends in the data.

Dynamic causal modelling is generally used to test hypotheses about the causal structure that generates data. This rests on Bayesian model comparison, where each hypothesis or model is scored using a (a variational bound) on model evidence. This enables one to find the best explanation for the data at hand that has the greatest predictive validity. This follows because cross-validation accuracy goes hand-in-hand with model evidence: in other words, maximising model evidence precludes over fitting by minimising complexity—and ensures generalisation to new data^6,17,20^. This is particularly prescient for epidemiological modelling because ‘new data’ pertains to the future, which means generalisation corresponds to predictive validity. Using models for forecasting that have not been subject to appropriate Bayesian model selection may have poor predictive validity because they overfit, if too complex, or underfit, if not sufficiently expressive. An example is provided in Fig. 4.

**Figure 5:**
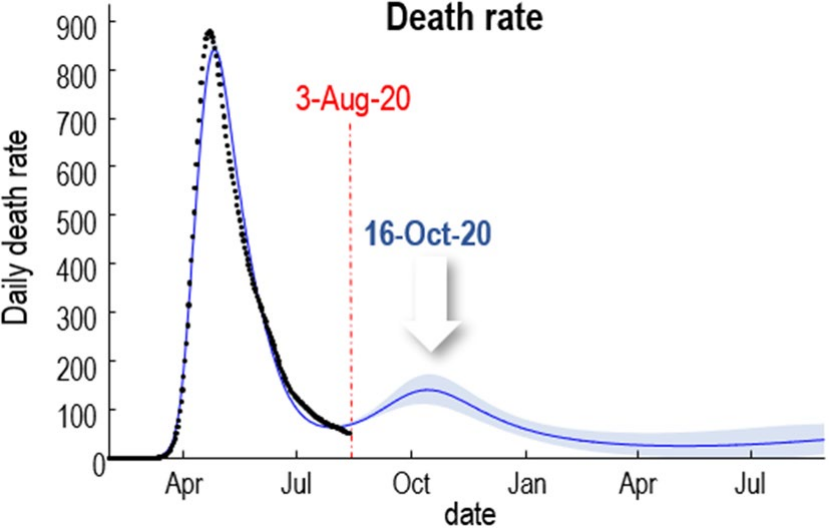
Dynamic causal modelling predictions of a second wave before any increase in confirmed cases in early August. This figure is taken from an internal report on the causes of second waves^13^.

Figure 4 illustrates a failure of forecasting with epidemiological (transmission) models^5^ that have not been optimised using variational model comparison. The left panels show the forecasts from the MRC Biostatistics Unit at the University of Cambridge, UK, shortly before and after the first peak of a second surge. These projections are based on screen grabs from the dashboard^4^ on appropriate days. The right panels show the equivalent predictions using dynamic causal models (Taken from the dashboard at^21^) following Bayesian model selection; specifically, Bayesian model reduction based upon a variational free energy evidence bound. The first MRC forecast on 11-Nov-20 predicted that the number of deaths each day would rise exponentially and “is likely to be between 380 and 610 on the 21st of November”. In fact, death rates peaked on 9 November at 398 (7-day average, evaluated on 20-Nov-20), as predicted by dynamic causal modelling. Crucially, the dynamic causal modelling predictions were consistent before and after the peak. Conversely, the MRC predictions had no predictive validity, forecasting opposite trends before and after the peak (indicated with the red ellipse). Furthermore, dynamic causal modelling predicted this peak before an upsurge in confirmed cases, albeit with a smaller amplitude and 3 weeks prematurely (see Fig. 5).

**Figure 6:**
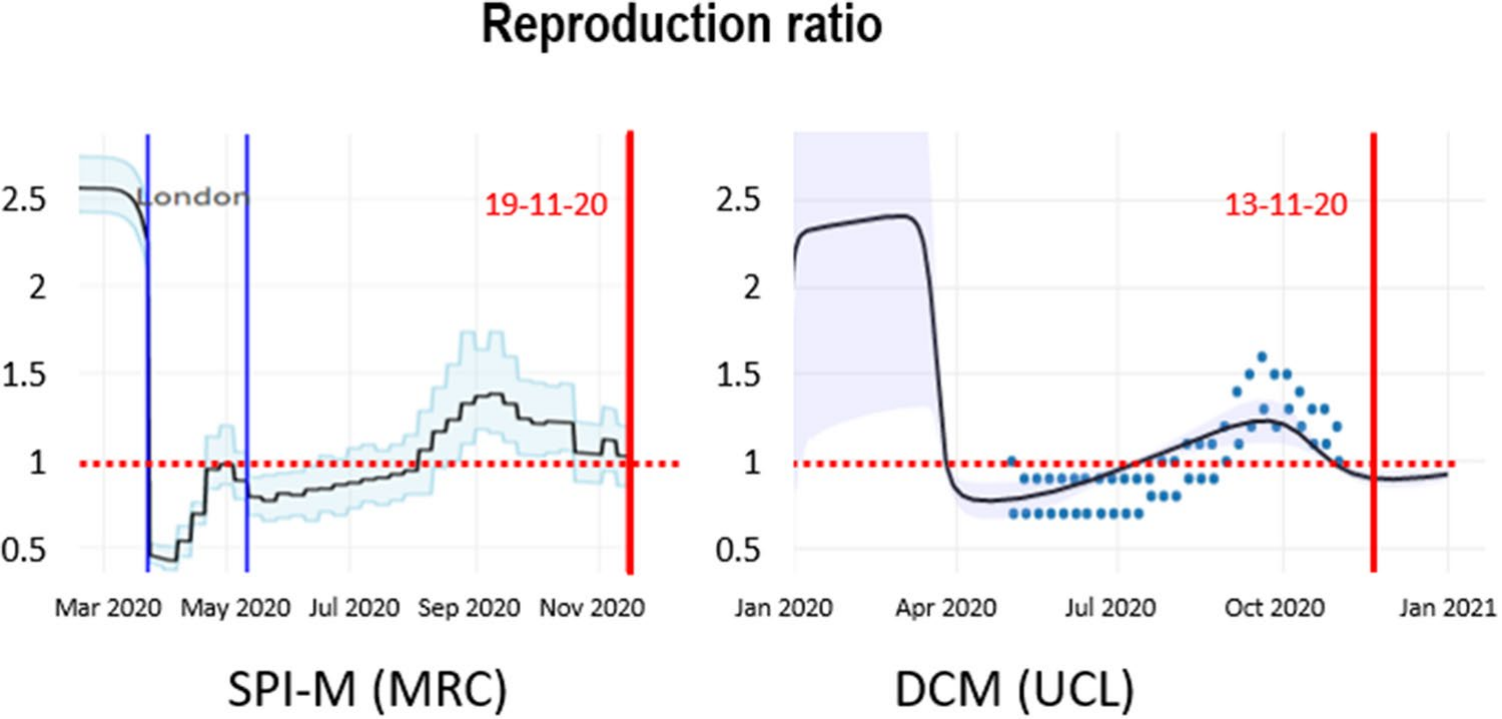
Estimates of the reproduction ratio from the MRC Biostatistics Unit (left panel) and dynamic causal modelling (right panel). The black lines correspond to the best (posterior) expectations and the shaded area correspond to credible intervals. The blue dots superimposed over the dynamic causal modelling estimates report the ranges of consensus values reported by the government^22^. These have been plotted 2 weeks before the end date of the reporting period for each pair. The vertical red lines indicate the date of estimation in mid-November.

An important application of nowcasting is to estimate the reproduction ratio (or R-number). As noted above this is often used as a point of reference for evaluating when various mitigations should be considered. However, when estimated using models that have not been optimised, estimates of things like the reproduction ratio can, in principle, become inaccurate and biased. An example is shown in Fig. 6. The left panel shows the reproduction ratio in terms of an expected value (black line) and credible intervals from the MRC Biostatistics Unit at the University of Cambridge (for London). The right panel shows the upper and lower intervals (blue dots) based upon a consensus of several modelling groups that constitute the SPI-M^15^ (for the United Kingdom).

In both instances, the reproduction ratio was estimated to be above one at the time the number of new infections peaked in the United Kingdom. This is mathematically impossible because the R-number should be exactly one at the time of peak incidence. Conversely, the estimates based on dynamic causal modelling (see Fig. 6 and Supplementary Information) suggest that the reproduction ratio fell below one about 3 weeks before the peak in death rates (black line in the right panel). The dynamic causal modelling estimates have a similar amplitude to the MRC and consensus (SPI-M) estimates; however, the latter lag the former by two weeks. In short, retrospective estimates that are used to motivate various nonpharmacological interventions—and assess their relative impact—may not be apt for guiding time-sensitive decisions.

## Discussion

The main contribution of DCM for COVID-19 is to place conventional (SEIR) models in a larger model of sociobehavioural responses and, crucially, models of how people seek out tests or healthcare. This involves having a multifactorial model, in which SEIR models constitute one of several factors. This enables the DCM to generate a wide range of measures and outcomes, and therefore use more empirical (real-world) kinds of data to optimise the model and its predictive validity. We have illustrated this anecdotally, by comparing the predictions of models that have and have not been optimised with respect to their marginal likelihood.

A key consideration in optimising the model—and implicitly its predictive validity—is getting the coarse graining or expressivity of the model right. Because the model evidence can be written as accuracy minus complexity—where complexity scores the degrees of freedom used to provide an accurate prediction of the data—the optimal model depends upon the data it is trying to explain. This means model evidence (or variational free energy) can be used to evaluate models with different degrees of freedom. For example, one could consider models in which social distancing and mask wearing were sensitive to different variables (e.g., social distancing increased with hospitalisation rates, while mask wearing increased with prevalence). If this more complex model had greater evidence then a simpler model—in which social distancing and mask wearing were subsumed into an effective contact rate (i.e., number of effective contacts per day, as in the current model)—then this would licence the distinct modelling of social distancing and mask wearing.

One might ask, what are the relative advantages of DCM in relation to alternative approaches; for example, machine learning (ML). Two key differences between DCM, in relation to ML are: (i) an explicit formulation in terms of a generative model and (ii) uncertainty quantification. A generative model is necessary if one wants to use the optimised model (and parameters) for scenario modelling: e.g., Refs.^23,24^. For example, if one wanted to ask how relaxing restrictions on contact rates would affect viral spread, one can change a parameter that couples prevalence to contact rates and predict the consequences. This would not be possible using ML, because there is no explainable or interpretable parameterisation in terms of prevalence or contact rates^25^. Having said this, if certain parts of the model to not need to be interpreted they could be optimised using ML. A nice example of this can be found in Ref.^26^, who used long short-term memory (LSTM) to estimate the (uninterpretable) parameters of the likelihood mapping between the (interpretable) latent states of a dynamic behavioural model and (observable) outcomes.

The ability of DCM to quantify uncertainty (c.f., confidence accumulation) means that any predictions or scenario modelling can be equipped with credible intervals in a straightforward fashion: c.f.^27,28^. This would not be possible using ML that, usually, does not learn probability densities or beliefs. Conversely, DCM deals with probability densities explicitly, using variational Bayes.

Variational Bayes rests on the calculus of variations, as applied to something called a mean field approximation; namely, a factorisation of the posterior density over unknown variables that lends it a known and tractable functional form. This functional form allows one to evaluate a lower (variational free energy) bound on model evidence (a.k.a., marginal likelihood) that is necessary for model comparison. The particular mean field approximation used in this DCM was straightforward; namely, a factorisation into two multivariate Gaussian distributions over (i) the parameters of transitions among states (and likelihood model) and (ii) the precision of random fluctuations in the data. Because the posterior densities are over the log of the respective parameters, the (nonnegative, scale) parameters have, effectively, a lognormal distribution.

In DCM, the (sufficient statistics of the) posterior densities are optimised using a (Newton) gradient ascent on variational free energy (a.k.a., evidence lower bound) where, crucially, the Gaussian form of the posterior enables the gradients to be evaluated analytically. This is known as Variational Laplace^10^. Practically, the advantage of Variational Laplace is its computational efficiency, in relation to alternative sampling schemes: c.f.^29^: i.e., minutes as opposed to hours. More importantly, the variational free energy is considered to be a much better approximation to model evidence than approximations based on sampling schemes (e.g., Metropolis Hastings), such as the Bayesian Information Criteria, harmonic means (please see Ref.^30^ for discussion.), et cetera^6,31^.

One disadvantage of variational procedures is known as the overconfidence problem. In other words, in using a mean field approximation, the marginal posterior densities over the factors are generally too precise, because they preclude conditional dependencies^17^. To accommodate this, one can scale the posterior covariances of the parameters, so that the 90% credible interval of the posterior predictive density contains about 90% of the empirical data points. In the present setting, this involved scaling the posterior standard deviation by a factor of eight.

## Epilogue

This report focuses on the model structure at a particular point during the COVID-19 pandemic. This model was subsequently used to produce weekly dashboard summaries of epidemiological trajectories and forecasts in the UK^3^. The accompanying dashboard contains links to weekly reports that list changes to the model. We have deliberately not updated the examples—or description of the model—in this article, which can be read as the initial basis for subsequent modelling.

However, it is natural to ask whether this kind of modelling proved useful over the ensuing months. The answer is yes and no. Up until the submission of the current report, DCM showed a high degree of predictive validity, widely acknowledged as providing the most accurate predictions of hospitalisation and morbidity during the first wave^1^. However, it failed to predict the course of the second wave in the UK, after the emergence of the alpha variant. This speaks to the importance of Bayesian model selection and how the best model changes as more data becomes available. In this case, the DCM described above could not model increases in transmission risk. With the advent of the alpha variant, models that included fluctuating transmission risk and seasonality effects (modelled with temporal basis functions) afforded greater model evidence and therefore replaced the current model^32^. Subsequently, further model updates were required to accommodate vaccination, the rollout of lateral flow device testing at scale, age stratified data, and so on.

Anecdotally, DCM predictions of prevalence, hospitalisation and mortality rates in the UK outperformed projections based upon conventional epidemiological modelling; especially worst-case scenario modelling. This is unsurprising because the objective of DCM is to predict the most likely outcomes, given the data at hand. Conversely, the objective of reasonable worst-case scenario modelling is to project outcomes under various predefined scenarios that may or may not come to be. We hope to quantify the predictive validity of DCM retrospectively, using global data, in the forthcoming year.

## Conclusion

In dynamic causal modelling, everything is optimised with respect to the marginal likelihood or evidence for a model, as scored by a variational free energy or evidence bound. This has the important consequence that the best model is a function of the data at hand. In turn, this means that the best model at the beginning of the epidemic is not the best model halfway through. As more data becomes available, the model needs to become more expressive (or complex) in order to provide an accurate account of these data. The complexity depends upon how tight the priors are over the model’s free parameters. There is an optimal complexity that, in conjunction with the accuracy of fit, subtends model evidence.

This optimal complexity can be identified using Bayesian model comparison. In other words, the model is defined in terms of which parameters are allowed to vary and which are, a priori, more constrained. This model optimisation is itself an adaptive and ongoing process that can, in principle, continue as long as data keeps arriving. As noted above, although Bayesian model comparison has been used throughout the epidemic, there is no guarantee that the basic form of the model—or its coarse graining—is necessarily the best. This would depend upon an exhaustive search of the model space, which is a difficult problem. A problem that may be addressed when the epidemic enters its endemic phase.

## Supplementary Information

Supplementary Information.

## Data Availability

All data analysed during this study are available for academic research purposes from the sites listed in the fourth column of Table S1.

## Code availability

The figures in this report can be reproduced using annotated (MATLAB) code available as part of the free and open source academic software SPM (https://www.fil.ion.ucl.ac.uk/spm/), released under the terms of the GNU General Public License version 2 or later. The routines are called by a demonstration script that can be invoked by typing >  > DEM_COVID_UK at the MATLAB prompt. At the time of writing, these routines are undergoing software validation in our internal source version control system—that will be released in the next public release of SPM (and via GitHub at https://github.com/spm/). In the interim, please see https://www.fil.ion.ucl.ac.uk/spm/covid-19/.

## References

[1] Friston KJ (2020). Dynamic causal modelling of COVID-19. Wellcome Open Res..

[2] Friston KJ (2020). Second waves, social distancing, and the spread of COVID-19 across America. Wellcome Open Res..

[3] WCHN. https://www.fil.ion.ucl.ac.uk/spm/covid-19/forecasting/. Accessed 18 July 2022.

[4] MRC-BSU. https://www.mrc-bsu.cam.ac.uk/tackling-covid-19/nowcasting-and-forecasting-of-covid-19/. Accessed 18 July 2022.

[5] Birrell PJ (2020). Real-time nowcasting and forecasting of COVID-19 dynamics in England: The first wave?. medRxiv.

[6] Penny WD (2012). Comparing dynamic causal models using AIC, BIC and free energy. Neuroimage.

[7] Hoeting JA (1999). Bayesian model averaging: A tutorial. Stat. Sci..

[8] Friston, K., Parr, T., Zeidman, P. *Bayesian model reduction.* arXiv preprint arXiv:1805.07092 (2018).

[9] Friston K, Penny W (2011). Post hoc Bayesian model selection. Neuroimage.

[10] Friston K (2007). Variational free energy and the Laplace approximation. Neuroimage.

[11] Beal, M. J. *Variational Algorithms for Approximate Bayesian Inference.* PhD. Thesis, University College London, 2003.

[12] Winn J, Bishop CM (2005). Variational message passing. J. Mach. Learn. Res..

[13] Friston, K. J. & Billig, A. J. *What causes second waves?* 2020. https://www.fil.ion.ucl.ac.uk/spm/covid-19/TR5_Second_Wave.pdf. Accessed 18 July 2022.

[14] ZOE. https://health-study.joinzoe.com/data#levels-over-time. Accessed 18 July 2022.

[15] SPI-M. https://www.gov.uk/government/groups/scientific-pandemic-influenza-subgroup-on-modelling. Accessed 18 July 2022.

[16] GOV-UK, https://www.gov.uk/government/news/prime-minister-announces-new-national-restrictions. Accessed 18 July 2022.

[17] MacKay DJC (2003). Information Theory, Inference and Learning Algorithms.

[18] Royal-Society. https://royalsociety.org/-/media/policy/projects/set-c/set-covid-19-R-estimates.pdf.

[19] Bar-On, Y. M., *et al.**A quantitative compendium of COVID-19 epidemiology.* arXiv (2020).

[20] Hochreiter S, Schmidhuber J (1997). Flat minima. Neural Comput..

[21] WCHN. https://www.fil.ion.ucl.ac.uk/spm/covid-19/dashboard/. Accessed 18 July 2022.

[22] GOV-UK. 2022. https://www.gov.uk/guidance/the-r-number-in-the-uk#contents. Accessed 18 July 2022.

[23] Gandolfi D (2020). Dynamic causal modeling of the COVID-19 pandemic in northern Italy predicts possible scenarios for the second wave. medRxiv.

[24] Wu JT, Leung K, Leung GM (2020). Nowcasting and forecasting the potential domestic and international spread of the 2019-nCoV outbreak originating in Wuhan, China: A modelling study. Lancet.

[25] Marcinkevičs, R. & Vogt, J. E. *Interpretability and Explainability: A Machine Learning Zoo Mini-tour*. arXiv:2012.01805. (2020).

[26] Miad Zandavi, S., Rashidi, T. H., Vafaee, F. *Forecasting the Spread of Covid-19 Under Control Scenarios Using LSTM and Dynamic Behavioral Models*. arXiv:2005.12270. (2020).

[27] Cooper, G. A method for using belief networks as influence diagrams. In *In Proc. of the Conference on Uncertainty in Artificial Intelligence*. (1988).

[28] Davidson L, Davidson L (1999). Uncertainty in economics. Uncertainty, International Money, Employment and Theory: Volume 3: The Collected Writings of Paul Davidson.

[29] Sengupta B, Friston KJ, Penny WD (2014). Efficient gradient computation for dynamical models. Neuroimage.

[30] Radford Neal Blog. https://radfordneal.wordpress.com/2008/08/17/the-harmonic-mean-of-the-likelihood-worst-monte-carlo-method-ever/. Accessed 18 July 2022.

[31] Fourment M (2019). 19 Dubious ways to compute the marginal likelihood of a phylogenetic tree topology. Syst. Biol..

[32] Friston, K. & Costello, A. *What we have learned from the second covid-19 surge?* (2020). https://blogs.bmj.com/bmj/2020/12/08/karl-friston-and-anthony-costello-what-we-have-learned-from-the-second-covid-19-surge/. Accessed 18 July 2022.

