# Supplementary material for: Dynamic causal modelling of COVID-19 and its mitigations
Source: Sci Rep. 2022 Jul 20;12:12419. doi: 10.1038/s41598-022-16799-8 (PMC9298167; doi:10.1038/s41598-022-16799-8)
Supplement: Supplementary file 1 — Supplementary Information. [file 41598_2022_16799_MOESM1_ESM.docx]

# Supplementary Information

# for

Dynamic causal modelling of COVID-19 and its mitigations

Karl J. Friston^1^, Guillaume Flandin^1^, Adeel Razi^1,2,3^

^1^*The Wellcome Centre for Human Neuroimaging, University College London, UK*

*^2^Turner Institute for Brain and Mental Health & Monash Biomedical Imaging, Monash University, Clayton, Australia*

*^3^CIFAR Azrieli Global Scholars Program, CIFAR, Toronto, Canada*

***E-mails****: Karl Friston,* [*k.friston@ucl.ac.uk*](mailto:k.friston@ucl.ac.uk)*; Guillaume Flandin, [g.flandin@ucl.ac.uk](mailto:g.flandin@ucl.ac.uk); Adeel Razi,* [*adeel.razi@monash.edu*](mailto:adeel.razi@monash.edu)

# Supplementary Information

This supplementary information describes the form of the generative model. The precise details of the implicit likelihood model and priors can be found in the annotated MATLAB scripts that generated the figures in this report. Details about the standard variational inversion of this model can be found in ^1^. The trajectory of the epidemic has called for an expressive DCM that can fit a large family of trajectories in different outcome modalities. At first glance, this expressivity may be confused with over-parameterisation. However, the effective degrees of freedom—or effective number of parameters—depend sensitively upon prior probability densities that themselves have been optimised using Bayesian model reduction. In other words, some parameters have uninformative priors and can be considered free parameters, while others have relatively tight or informative priors. The effective number of parameters corresponds to model complexity; namely, Kullback-Leibler divergence between the posterior and the prior. This effectively counts the number of parameters that are used to explain the data.

At the time of writing, the dynamic causal modelling (DCM) has about 40 parameters that parameterise 400 differential or update equations. This may sound like a large number; however, these update equations inherit from fairly common-sense assumptions about transitions among latent states. These structural assumptions, in combination with the prior densities, constitute the assumptions made by the model. At no point do we assume any particular parameter is known: every model parameter is equipped with a greater or lesser posterior uncertainty, expressed by the priors in Table 1.

The basic form of the DCM is built upon a Master equation ^21^ that describes the discrete updates of the probability over the latent states of the model , day by day:

$\begin{matrix} \vec{p}_{\tau+1}=P\cdot\vec{p}_{\tau} \\ \vec{p}=vec(p) \end{matrix}$ (S1

This equation can also be expressed as a set of ordinary differential equations by noting the following equivalence:

(S2

Here, *J* plays the role of the Jacobian of the density dynamics and would have the continuous differential equation form used in most conventional modelling. However, we will stick to the discrete form using the Master equation, based upon a large probability transition matrix *P*. This matrix can be factorized into transitions among the states of each of the four factors (denoted by superscripts). In what follows, we will use $P_{\circ,\bullet,\bullet,\bullet}$ to denote the probability transitions within the first factor states, $P_{\bullet,\circ,\bullet,\bullet}$ denotes probability transitions within the second factor, and so on. Here, the dot stands in for of all levels of the factor corresponding to the order of the index. Similarly, $p_{\circ,\bullet,\bullet,\bullet}$ (note the lower-case *p*) represents the marginal distribution having averaged over the factors denoted by the black dots. For example, $p_{1,\bullet,\bullet,\bullet}$ is the probability of being in the first state of the location factor, marginalised over all other factors. The matrix factorization is as follows:

$\begin{matrix} P=P^{(1)}\cdot P^{(2)}\cdot P^{(3)}\cdot P^{(4)} \\ \\ P^{(1)}=I_{4}\otimes\left[\begin{matrix} I_{2}\otimes P_{\circ,\bullet,1,\bullet} & & & \\ & I_{2}\otimes P_{\circ,\bullet,2,\bullet} & & \\ & & I_{2}\otimes P_{\circ,\bullet,3,\bullet} & \\ & & & I_{2}\otimes P_{\circ,\bullet,4,\bullet} \end{matrix}\right] \\ P^{(2)}=\ldots\\ \end{matrix}$ (S3

This means that we can build the model by considering transitions among states of each factor in turn and then compose these factor-specific probability transition matrices to build the Master equation above. This would be a simple procedure if the transitions among each factor did not depend upon each other. However, a key aspect of this sort of DCM is an inherent interdependency among the factors, in which the probability of moving from one state to another—within one factor—depends upon the probability distribution over the states of another factor. For example, the probability that I will move from an asymptomatic to a symptomatic state depends upon the prevalence of infection; namely, the probability that I am infected. These (second-order) dependencies can be expressed as probability transition matrices within each factor that are conditioned upon the levels of another. In what follows, we will go through the four factors describing the second-order dependencies and occasional third-order dependencies. Third-order dependencies mean that the influence of one factor on another depends upon a third factor. These high order dependencies need to be incorporated into the Master equation, after it is composed according to Equation S3.

To illustrate how this DCM is parameterised, we will now go through each of the factors in turn. The equations and underlying latent states (i.e., compartments) will be presented as figures, which are followed by intuitive descriptions. Please see the MATLAB code for one or two details that have been omitted for clarity. There are four factors, *location*, *infection*, *symptom*, and *testing*. These factors have four or five levels or states each.

### Location


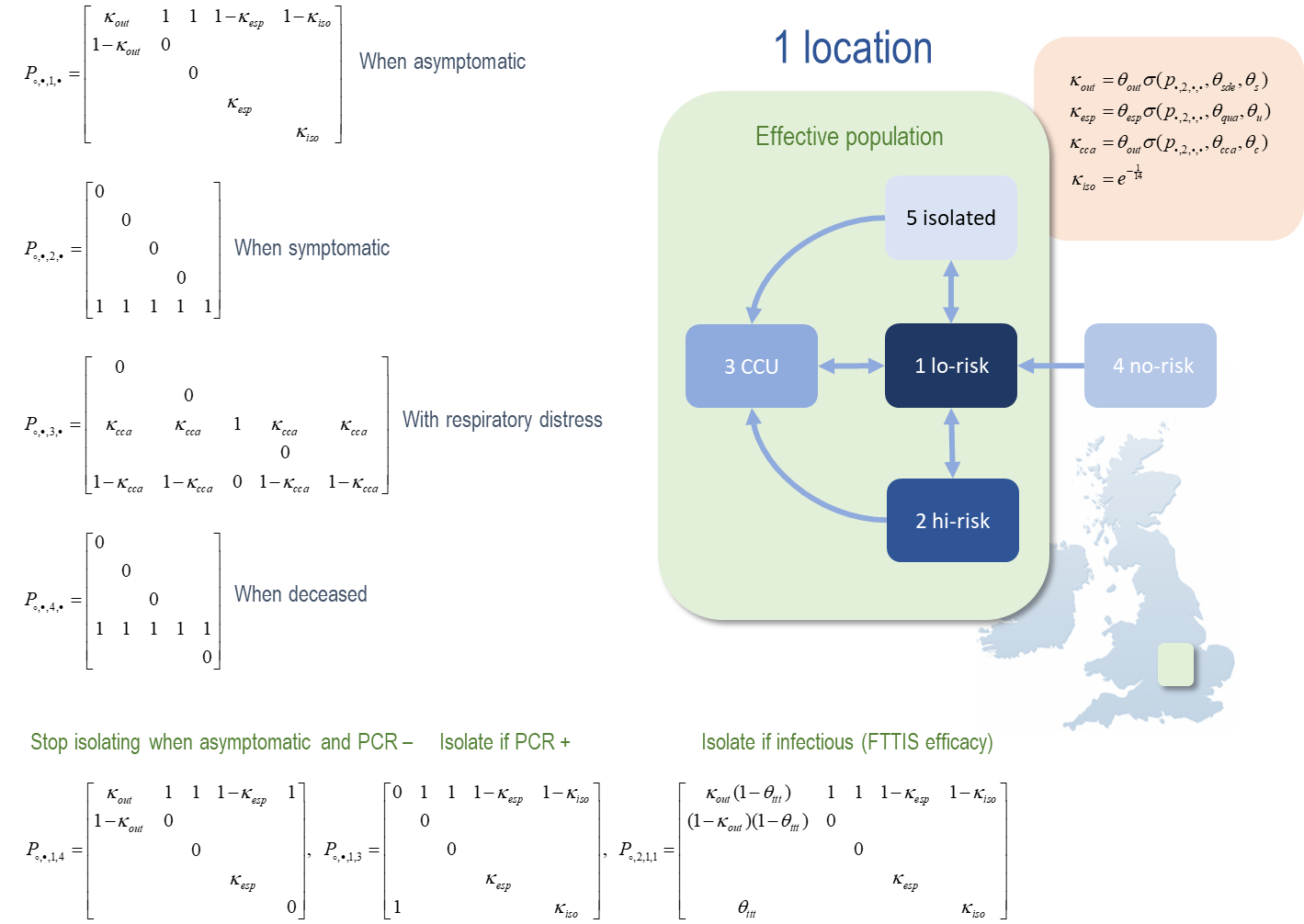


**Figure S1:** *location*

The location factor has five states, four of which constitute an *effective* or *affected* population that is a subset of the total or census population. The remainder of the population is assigned to a *no-risk* state. The four states of the effective population include a low and high-risk state. Here, risk refers to the probability of coming into contact with somebody who is infected. Low-risk could be being at home (in an affected area), while high-risk could be being at work or a football match (in an affected area). In addition to these two locations, one could be in critical care (requiring mechanical ventilation) or self-isolated. The transition matrices on the left of Figure S1 describe the transitions amongst these location states when *asymptomatic*, *symptomatic*, when severely ill (e.g., acute respiratory distress syndrome, *ARDS*) or when *deceased*. These four states are the levels of the *symptom* factor.

The transition matrices in Figure S1 show that when *asymptomatic*, I have a certain probability of leaving the low-risk (home) location and entering a high-risk (e.g., work) location. When at work, I will inevitably return home in the evening. When in a critical care unit (*CCU*), I will be discharged when and only when *asymptomatic*. In terms of moving from a low-risk state to *isolation*, I will stay in self-isolation for 14 days and return to a low-risk (e.g., domestic setting) after that time. Similarly, if I am *symptomatic*, I will always go into self-*isolation* and when *deceased*, go into a *no-risk* state. When in acute respiratory distress (*ARDS*) I will be taken to critical care (*CCU*) unless I am already there. However, there is also a possibility that the capacity of critical care has been exceeded—and I am not admitted—in which case, I will go into isolation.

The third-order dependencies are described in the lower panel of transition matrices (labelled with green text). For example, I will stop self-*isolating* provided I do not have symptoms and I am PCR negative. Similarly, if I am asymptomatic and test positive, then I will go into *isolation*. The third route to isolation models the efficacy of find test trace isolate and support (FTTIS). This is captured by the probability that I will go into isolation if I am (told by a contact tracer that I am) asymptomatic and infected.

The *no-risk* state (state number four) models a reservoir of the population that has yet to be affected by the epidemic. In this (simplified) description, one can only move from a no-risk to a low-risk state. Effectively, this models the spread of the virus through communities, thereby enlarging the effective population as time goes on. In a full implementation, there is also an efflux from the affected population (i.e., a low-risk state) back into a no-risk state. With this bidirectional exchange—between the effective and no-risk population, the size of the affected population can reach some equilibrium depending upon the relative rates of influx and efflux. We have omitted these effects to avoid visual clutter. In other words, the model in this figure just allows for a progressive increase in the size of the affected population or the number of communities that are exposed to the virus as time proceeds. A simplifying assumption here is that we have used (epidemic) model of a single region—of varying size—as opposed to a (pandemic) model of multiple regions described elsewhere^2^.

The format of the equations is reproduced in subsequent figures: $\kappa$generally refers to a rate constant, namely, the parameters of transition probabilities. The free parameters of the model parameterise these rate constants and are denoted by $\theta$ (the functional form of this parameterisation is provided in the pink boxes). For example, the probability that I will leave home is the product of a baseline probability of going to work times a decreasing sigmoid function of the prevalence of infection. The midpoint of this sigmoid function can be regarded as a soft threshold. In a similar way, the influx of people who have hitherto been in no-risk areas is a decreasing function of prevalence at a lower threshold. It is the disparity between these two (lockdown versus quarantine) thresholds that generates secondary and subsequent waves.

Heuristically, as the virus replicates within the effective population (i.e., the community into which a virus was introduced, such as a city) the lower threshold is crossed and the influx of people into the effective population falls. This can be mediated by travel restrictions, quarantine, or a cordon sanitaire. Within the effective population, prevalence continues to increase until the second threshold is crossed, and within-community distancing is realised through a decreased probability of leaving low-risk locations (i.e., ‘stay at home’). Community transmission is attenuated, and the prevalence of infection peaks and then falls as the community acquires a degree of population immunity. As prevalence falls below the lockdown threshold the community unlocks, and social distancing measures are relaxed: for example, the period after the first wave during the summer in the United Kingdom. However, as prevalence falls towards the lower threshold there is an influx from the no-risk locations as travel restrictions are relaxed (e.g., students returning to university), and population immunity is subsequently diluted. The virus then starts to propagate through the extended effective population, engendering a secondary wave.

This succession of between and within-community changes in population mixing and contact rates furnishes a simple model of viral spread throughout the total population, producing a succession of progressively attenuated waves. Note that there is a similar threshold function for entering the critical care state that models a limited capacity. We will see later that the only way to survive severe clinical consequences of infection (e.g., acute respiratory distress syndrome) is to be admitted to critical care. The final parameter determines how long one is in critical care, expressed as a time constant. These time constants can be regarded as expected dwell time or duration that one is in a particular state. This concludes our description of the location model, where transitions among different states depend primarily upon the symptom factor but also have third order dependencies on the infection and testing factors.

### Infection

Figure S2 uses the same format to detail transitions among different states of infection. Here, people start out in a *susceptible* state from which they can get *infected*. From the infected stated there are two routes to an absorbing state (c.f., the removed state of conventional SEIR models). One can either have a mild illness and move straight to a seronegative state ^22, 23, 24^ that may be associated with T-cell mediated humoral immunity ^23, 24, 25^. Conversely, one can have a more severe illness with viral shedding ^26^ and move to an infectious state, and then become seropositive (Ab+) for a period of time ^27, 28, 29, 30^. This period of time corresponds to loss of antibodies ^31^, parameterised with the appropriate time constant, causing a transition from seropositive to seronegative immunity (Ab-). The immunity is modelled by precluding a return from seropositive to susceptible, which could be relaxed if necessary.


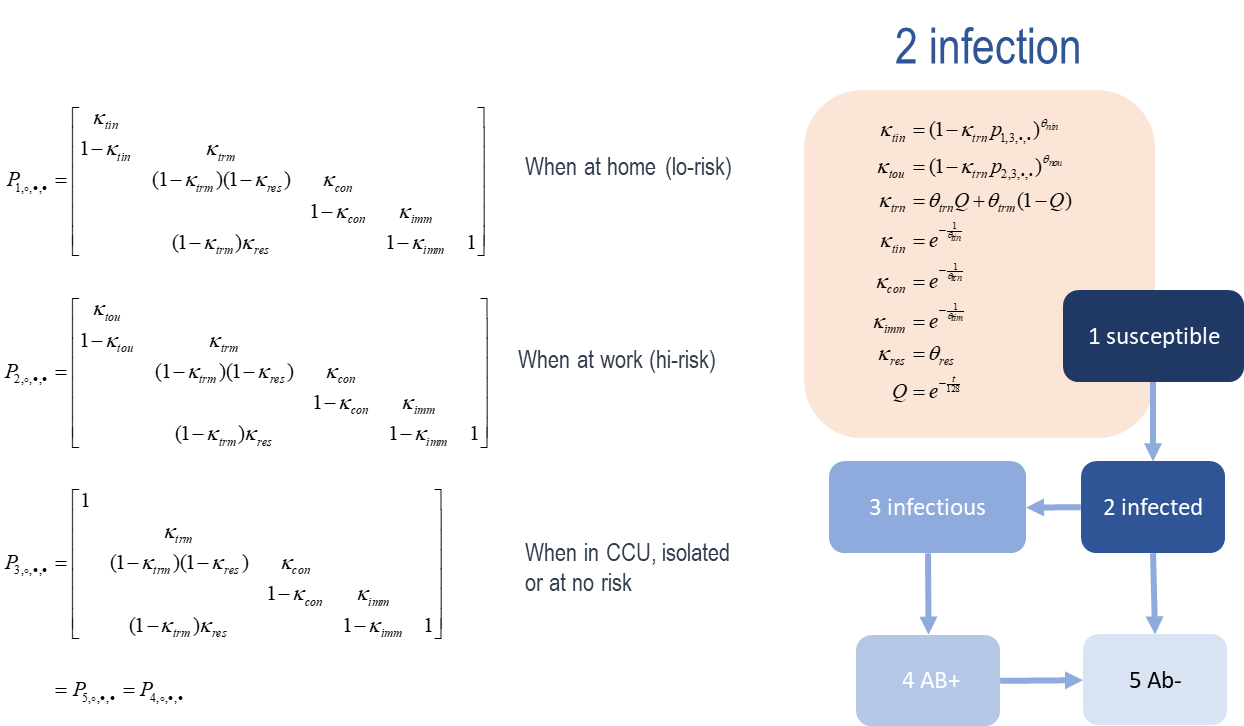


**Figure S2:** *infection*

The rate constants for this factor deal with the probability of becoming *infected*, parameterised in terms of different contact rates. More specifically, the probability of remaining *uninfected* is the probability of avoiding contagion raised to the power of the number of expected contacts per day. In turn, this depends upon whether you are in a low or high-risk situation—or indeed are isolated in CCU or are in a no-risk area. The probability of becoming infected per contact is itself a rate constant times the prevalence of infection in the respective area. In this model, the implicit transmission strength can change over time (either increasing or decreasing). The remaining rate constants are specified in terms of their expected time constants, while the proportion of people who seroconvert is specified by a free parameter. This parameter quantifies the overdispersion or heterogeneity of transmission ^32, 33^, in the sense that only a certain proportion of people ever become infectious after being infected. This proportion is estimated as a free parameter of the model—analogous to *k* in conventional models. This concludes our description of the infection model that depends on, and only on, the location factor.

### Symptoms


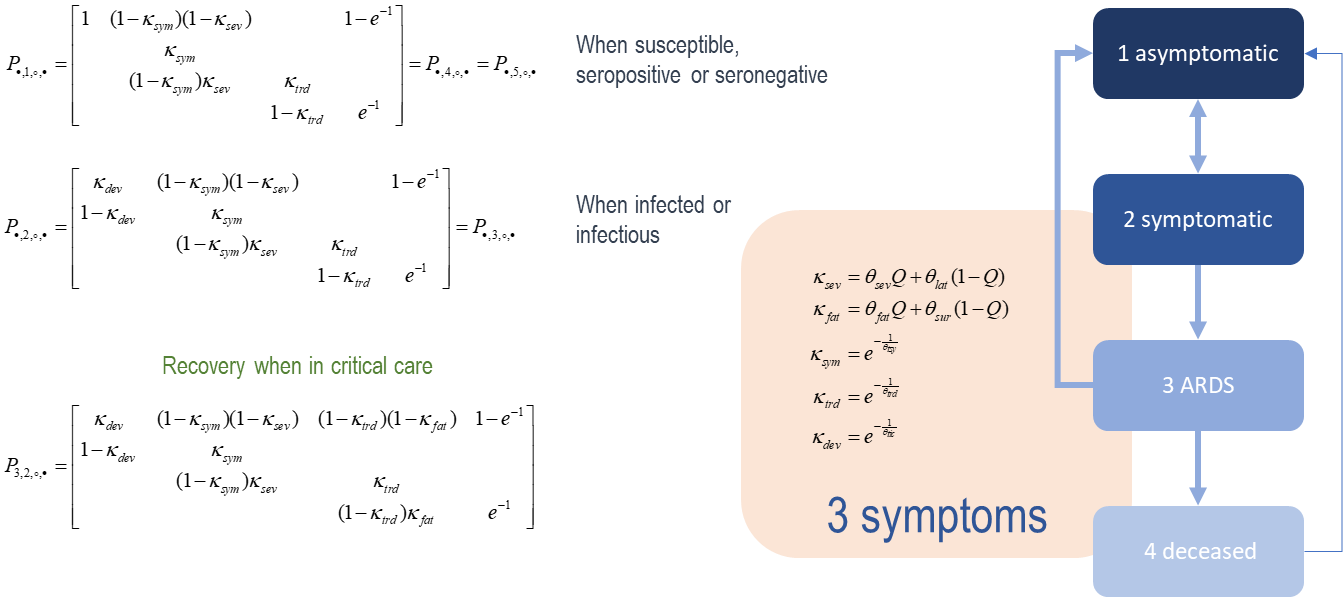


**Figure S3:** *symptoms*

The *symptom* or clinical factor has four states (see Figure S3). One can start in an *asymptomatic* state and then, consequent on being *infected*, can—with some probability—become *symptomatic*. A small proportion of *symptomatic* people will progress to potentially fatal respiratory distress and subsequently die, unless they are supported in critical care. The corresponding rate constants are again time-dependent, meaning that the probability of developing *ARDS* can change over different phases of the epidemic (either increase or decrease). Similarly, the probability of dying from *ARDS* can itself change with time, for example as treatments improve. The remaining parameters are specified in terms of the expected dwell times, here in terms of an incubation period, asymptomatic period, and a period of severe disease. Typically, these sum to about 20 days, which is the expected time between becoming infected and death.

### Testing

Testing has four states, starting with having never been tested (see Figure S4). One then has a test and *waits* for the results that can either be *positive* or *negative*. Having been tested, one then returns to the *not tested* state. The probability transition matrices are equipped with test sensitivity and specificity parameters, with negative tests when susceptible or no longer infected or infectious (i.e., *seropositive*, or *seronegative*). Conversely, when infected or infectious, there is a high probability of moving to the PCR positive state. Note that when not part of the effective population, one does not bother getting tested.


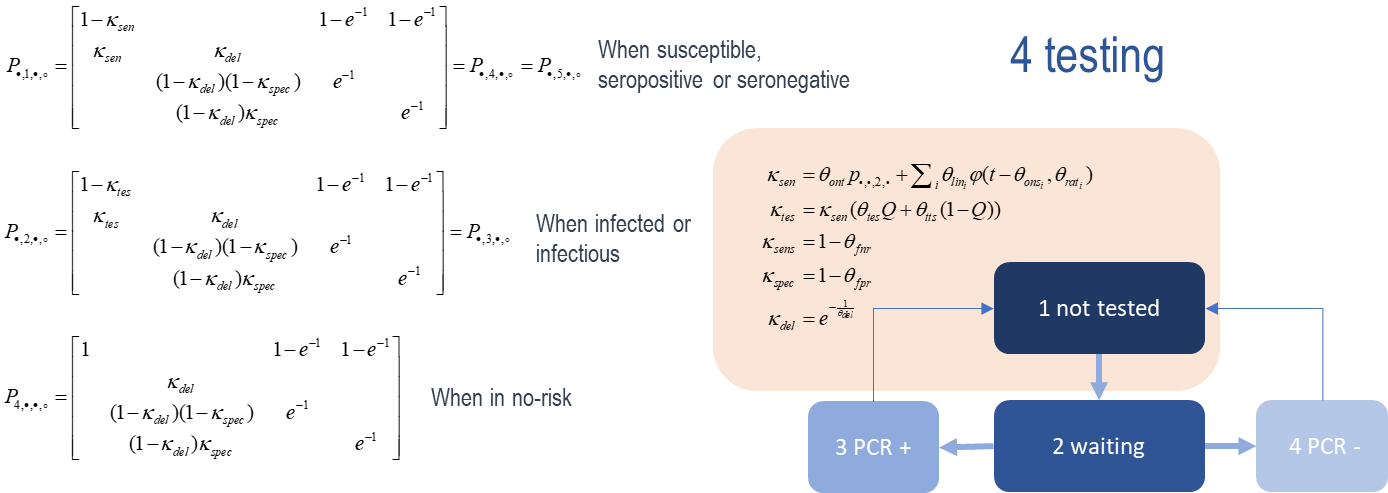


**Figure S4**: *testing*

The probability of submitting to a test depends upon testing capacity that is modelled with a linear mixture of increasing sigmoid functions—as various phases of testing are rolled out. In addition, the there is a component of test rates that reflects demand in terms of the prevalence of people who are symptomatic. Note further that self-selection bias is parameterised in terms of the relative probability of getting tested if infected, relative to not being infected. This testing bias can change with time (e.g., the relative number of pillar one and pillar two tests in the UK).

This concludes our discussion of the *testing* factor. The transitions among the various states above effectively constitute the prior probabilities that, when equipped with a likelihood model, complete the generative model. The likelihood model maps from the latent states above to observations by taking the expected outcome and adding a random effect.

### The likelihood model

The likelihood (a.k.a. observation) models used in this DCM are based upon simple counting statistics for large numbers. After a square root transform, the data can then be treated as a Gaussian variate with unit variance about the expected number. In practice, because the data are smoothed (which introduces serial dependencies among the data), DCM estimates the random observation effects under fairly informative hyperpriors. In other words, it adjusts the variance or precision of each data modality in proportion to the total number of observations based upon the residual sum of squares. Hyperpriors in this instance specify the prior beliefs about the variance of these residuals, automatically adjusting for the uncertainty in the data, relative to the priors above. In practice, the hyperprior expectation for the variance is set to the log of the summed observations for any kind of data. This automatically adjusts for sparse data with relatively few data points.

**Table S1**: outcomes and their expectations

| Outcome | Description | Likelihood model | Source | Units |
| --- | --- | --- | --- | --- |
| PCR cases (ONS) | Cases by specimen date (UK total) | $Np_{\bullet,\bullet,\bullet,3}$ | https://coronavirus.data.gov.uk/cases | number/day |
| Daily deaths (ONS) | Deaths within 28 days of positive test by date of death (UK total) | $Np_{\bullet,\bullet,4,\bullet}$ | https://coronavirus.data.gov.uk/deaths | number/day |
| Ventilated patients (ONS) | Patients in mechanical ventilation beds | $N\theta_{cc}p_{3,\bullet,\bullet,\bullet}$ | https://coronavirus.data.gov.uk/healthcare | number |
| PCR tests (ONS) | Number of confirmed positive, negative or void lab-based COVID-19 test results | $Np_{3,\bullet,\bullet,\bullet}$ | https://coronavirus.data.gov.uk/testing | number/day |
| Prevalence (ONS) | Estimate of the number of people testing positive for COVID-19 | $N(p_{\bullet,2,\bullet,\bullet}+p_{\bullet,3,\bullet,\bullet})$ | https://www.ons.gov.uk/peoplepopulationandcommunity/healthandsocialcare/conditionsanddiseases/datasets/coronaviruscovid19infectionsurveydata | number |
| Seropositive (GOV) | Number of people testing positive for COVID-19 antibodies | $Np_{\bullet,4,\bullet,\bullet}$ | https://www.ons.gov.uk/peoplepopulationandcommunity/healthandsocialcare/conditionsanddiseases/datasets/coronaviruscovid19infectionsurveydata | percent |
| Symptoms (KCL) | Number of people calculated to have COVID symptoms on each day | $N\theta_{sy}(p_{\bullet,\bullet,2,\bullet})$ | https://covid.joinzoe.com/data#levels-over-time | number |
| R-ratio (GOV) | The R number range for the UK |  | https://www.gov.uk/guidance/the-r-number-in-the-uk#contents | ratio |
| Transport (GOV) | Percentages of the first week in Feb-20 (Cars) | $\begin{matrix} \theta_{mo1}p_{2,\bullet,\bullet,\bullet}(p_{\bullet,1,\bullet,\bullet})^{\theta_{mo2}} \\ \times100 \end{matrix}$ | https://www.gov.uk/government/statistics/transport-use-during-the-coronavirus-covid-19-pandemic | percent |
| Work (Google) | Community Mobility Reports (Workplaces) | $\begin{matrix} \theta_{wo1}p_{2,\bullet,\bullet,\bullet}(p_{\bullet,1,\bullet,\bullet})^{\theta_{wo2}} \\ \times100 \end{matrix}$ | https://www.google.com/covid19/mobility/ | percent |

*Table S1 lists the outcomes used to invert the dynamic causal model. The predicted outcomes are a straightforward function of the marginal probabilities over various latent states. For example, the percentage of people who are seropositive is the proportion of people in the Ab+ state of the second factor multiplied by one hundred. Some predictions involve exponents. For example, the percentage of people at work is proportional to the probability of being in a high-risk location times the probability that any member of the population has yet to be infected, raised to a power. When this power is very small, this nonlinear term effectively disappears. All these expected outcomes are instantaneous functions of the probability, with the exception of the reproduction ratio that depends on the rate of change of prevalence.*

## Reproduction ratio

The effective reproduction rate is a fundamental epidemiological constant that provides a useful statistic that reflects the exponential growth of the prevalence of infection. There are several ways in which it can be formulated. For our purposes, we can generate an instantaneous reproduction rate directly from the time varying prevalence of infection as follows:

These expressions show that the reproduction rate reflects the growth of (the logarithm of) the proportion of people infected—and the period of being infectious. This number is formally related to the doubling time *T_d_*. Note that the reproduction rate is not an estimate in this scheme: it is an outcome that is generated by the latent causes or hidden states inferred by inverting (i.e., fitting) the model to empirical timeseries.
